# Intraspecific Contact Among White‐Tailed Deer: A Literature Review and Chronic Wasting Disease Case Study

**DOI:** 10.1002/ece3.73040

**Published:** 2026-02-12

**Authors:** Nathaniel H. Wehr, Kristin J. Bondo, Christopher S. Rosenberry, David Stainbrook, Bret D. Wallingford, W. David Walter

**Affiliations:** ^1^ Pennsylvania Cooperative Fish and Wildlife Research Unit The Pennsylvania State University University Park Pennsylvania USA; ^2^ Pennsylvania Game Commission Bureau of Wildlife Management Harrisburg Pennsylvania USA; ^3^ U.S. Geological Survey, Pennsylvania Cooperative Fish and Wildlife Research Unit The Pennsylvania State University University Park Pennsylvania USA

**Keywords:** association, encounter, *Odocoileus virginianus*, proximity, social interactions, transmission

## Abstract

White‐tailed deer ( *Odocoileus virginianus* ) are a valuable game mammal in the eastern United States necessitating detailed understanding of disease transmission. We conducted a literature review on intraspecific contact (i.e., interactions wherein disease transmission may occur) among deer. From 69 studies, we identified five themes underlying research on intraspecific deer contact: physical touch, social groups, spatial overlap, association rates, and social networks. Visual observations determined physical touch to be infrequent (< 2 touches/h) and indicated deer social groups were dependent on spatial dynamics of parturition and dispersal; most females remained with matriarchal family groups while males dispersed and formed bachelor groups. Assessed using global positioning system (GPS) monitoring, spatial overlap and association rates (i.e., instances of deer in close spatial–temporal proximity) were higher in correspondence to within‐group social dynamics, and between‐group scores were correspondingly low. Social network analyses indicated between‐group transmission may be driven by socially dominant males, often termed super‐spreaders (i.e., hosts infecting disproportionately high numbers of healthy individuals). We investigated these themes via a case study of deer infected with chronic wasting disease (CWD) in southcentral Pennsylvania, United States. We assessed spatial overlap and association rates using GPS monitoring data from 180 deer. Our results supported findings in the literature, showing strong correlations among spatial overlap, association rates, and correlated movements. Further, CWD‐infected deer exhibited similar association rates to deer in which CWD was not detected. Our literature review and case study indicate direct transmission of CWD and other diseases is likely greatest within social groups following seasonal behavioral dynamics and that between‐group transmission is likely driven by males via dispersal and mating interactions. Our results may be used to inform population management models with future work focused on high resolution spatial assessments of transmission in localized areas.

## Graphical abstract

We identified five themes underlying research on intraspecific contact among white‐tailed deer: physical touch, social groups, spatial overlap, contact rates, and social networks. We found white‐tailed deer infected with chronic wasting disease exhibited similar rates of intraspecific contact as those without infections.

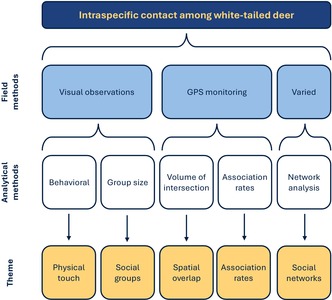

## 1 Introduction

Estimation of disease transmission rates is dependent on the proportion of the population susceptible to the disease, intraspecific contact rate (i.e., the frequency of intraspecific interactions wherein disease transmission may occur over time), and pathogen transmission probability during intraspecific interactions (McCallum et al. 2001; White et al. 2017). Detailed, species‐specific contact rate estimations are therefore crucial to disease modeling and, by extension, wildlife management (Tompkins et al. 2011; Joseph et al. 2013). In this context, direct contact occurs when two individuals are in close spatial–temporal proximity while indirect contact occurs when individuals are in close spatial proximity but distal temporal proximity (White et al. 2017). Though imperfect (Farine 2015), association rates (i.e., the frequency with which two individuals are observed as spatially proximate) are often used as a proxy for contact rates because interactions cannot be adequately assessed. We focused our review on direct intraspecific contact that may facilitate disease transmission, though we reference research examining indirect contact and using association rate proxies.

Common field methods used to assess intraspecific contact include visual observations, proximity loggers, and very high‐frequency (VHF) or global positioning system (GPS) monitoring devices (White et al. 2017). The spatial–temporal scales at which contact rates can be assessed constrain each method (Cross et al. 2012). For example, visual observations of meerkats ( *Suricata suricatta* ) were used to assess bovine tuberculosis ( *Mycobacterium bovis* ) transmission but were limited to crepuscular events outside burrows (Drewe 2009). Difficulty assessing whether and when individuals became infected in the field also constrains these studies (White et al. 2018). Association rates were calculated using GPS‐monitored wild boar ( *Sus scrofa* ) (Podgórski et al. 2018) and Cape buffalo ( *Syncerus caffer caffer* ) (Wielgus et al. 2021) in the context of disease transmission, but the disease status of monitored individuals was not assessed, exemplifying this shortcoming.

Examinations of contact rates among white‐tailed deer ( *Odocoileus virginianus* ; hereafter, deer) are important to applied management objectives given their societal value and disease transmission potential. Deer inhabit most of North America (Heffelfinger 2011; Gallina and Lopez Arevalo 2016) and are the predominant species of big game in the eastern United States (Hewitt 2015). Deer are hunted for recreation, subsistence, and as important components of Indigenous cultural autonomy (Conover 2011; Parlee et al. 2021). Economically, deer hunting and viewing is valued at > $16 billion annually, though difficult to isolate from other big game, greatly surpassing the nearly $5 billion in losses generated by deer–vehicle collisions and other human–deer conflict (Conover 2011; Hewitt 2015). Deer mortality is the most well‐studied of any species globally (Hill et al. 2019b, 2019a), and disease accounts for around 4% of deer mortality (Wehr et al. 2025a, 2025b), with much higher proportions in areas lacking predators (Dion 2018; Haus et al. 2020). Deer are also significant reservoirs for diseases affecting livestock and humans, which may worsen as deer populations increase in suburban and exurban landscapes (Lavelle et al. 2016; Kuchipudi et al. 2022).

The disease of greatest concern to deer health is chronic wasting disease (CWD). An always fatal transmissible spongiform encephalopathy (Williams et al. 2002), CWD is the only prion disease known to affect wildlife (Pritzkow 2022). In the United States and Canada, CWD has been detected among all species of endemic cervids across 36 states and 4 provinces (USGS 2025). Monitoring and applied management of CWD costs states an average of $500 thousand annually (Thompson and Mason 2022; Thompson et al. 2023). CWD prions are excreted through saliva, blood, mucosal tissue, urine, and feces and remain present on infected carcasses (Miller et al. 2004; Haley et al. 2011; Kramm et al. 2017). When deer populations are newly infected with CWD, direct contact is likely the primary pathway for CWD transmission until a sufficient environmental reservoir of CWD prions is accumulated (Almberg et al. 2011; Kjær and Schauber 2022).

Contact rates are vital to modeling disease transmission and incorporating disease dynamics into applied deer management (Joseph et al. 2013; White et al. 2017). Our objective was to comprehensively assess direct intraspecific contact among deer in this context. Specifically, we addressed the questions: what aspects of contact rates have previously been studied among deer; what field and analytical methods were most commonly applied to estimations of contact rates; which methods are most useful given current technological capabilities; and do these methods adequately assess disease transmission risk? We addressed these questions by conducting a literature review of intraspecific contact among deer. We then used association rates as a proxy to estimate contact rates among GPS‐monitored deer in a CWD management area as a complementary case study addressing an identified knowledge gap. We concluded by identifying future research priorities applicable to management‐driven disease transmission models.

## 2 Literature Review

### 2.1 Search, Screening, and Extraction

We conducted a literature review on intraspecific contact among deer. We conducted our first search during July 2024 using the Boolean operator AND to combine the search term “white‐tailed deer” with “contact*,” “disease transmission,” “proximity,” “social interaction*,” and “spatial distribution*” each individually. We conducted our second search during March 2025 using the Boolean operator AND to combine “white‐tailed deer” with “behavior*,” “group size,” “spatial overlap,” “contact rate*,” and “social network*” each individually. In both searches, we used the Google Scholar and Web of Science search engines, where an “*” denoted a partial term (e.g., “contact*” = “contact,” “contacts,” “contacted,” OR “contacting”). We did not limit either search to any time period or geographic region. The combinations of search terms and search engines resulted in 20 distinct search result sets. We additionally considered publications cited by or citing studies deemed relevant during the screening process using a snowball approach (Webster and Watson 2002; Wohlin 2014).

We screened the first 100 results of each set using the title, abstract, and body of each publication; we limited our effort to the first 100 results due to decreasing relevance. We considered peer‐reviewed journal articles, theses/dissertations, book chapters, pre‐prints, and conference proceedings for inclusion in our review, and we did not exclude any relevant search results regardless of the format. We included studies of wild deer and excluded those using captive deer or simulated data as unrepresentative of wild populations. We included only studies providing quantification or analytical estimation of contact rates (or appropriate proxies) and excluded qualitative or descriptive studies. We included studies discussing direct and indirect contact as well as studies not discussing results in the context of disease transmission. In cases of duplicated information (e.g., a thesis and a resulting peer‐reviewed journal article), we prioritized peer‐reviewed sources unless relevant information was provided only in the alternative.

**FIGURE 1:**
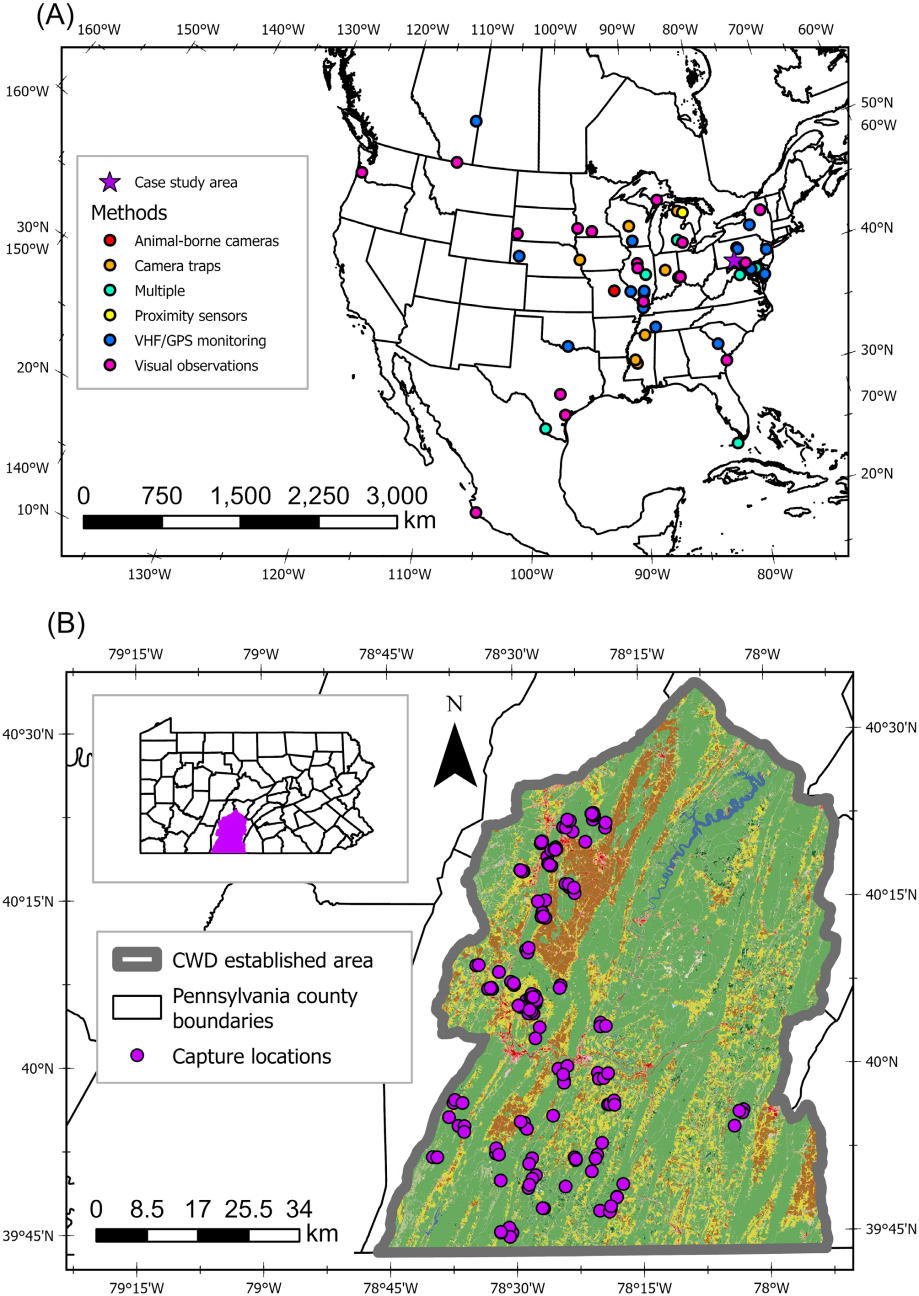
Locations of white‐tailed deer ( *Odocoileus virginianus* ) intraspecific contact studies across North America (Panel A) and capture site locations for our case study area in southcentral Pennsylvania, United States (Panel B). Methods for each study are labeled by colored points, with our case study area indicated by a purple star (Panel A). Within our case study area (indicated in purple in the inset map of Pennsylvania counties), capture locations of global positioning system‐monitored deer are labeled as purple points (Panel B). Land cover within the case study area used the 2019 National Landcover Database (Dewitz and USGS 2021). The most common landcover types were deciduous forest (61.5%; green), pasture (14.2%; yellow), cultivated crops (9.2%; brown), and anthropogenic development (7.0%; red).

Our screening and exclusion process produced 69 studies published during 1966–2025 examining deer ecology relevant to intraspecific contact. Three studies were identified exclusively via the snowball process. All remaining studies were identified in at least 1 of the 20 search sets (mean = 3.7 search sets, standard deviation [SD] = 3.0, range = 1–13). Most (*n* = 66) studies occurred in the contiguous United States, with the greatest concentration in the Midwest region: 15 in Illinois and 6 in Michigan (Figure 1A). Alberta hosted the only two studies in Canada, and one study was in western Mexico. We included two studies examining Columbian white‐tailed deer (*O. v. leucurus*) and one study each of Sinaloa white‐tailed deer (*O. v. sinaloae*) and Key deer (*O. v. clavium*); no other studies specified subspecies. Employed field methods included GPS or VHF monitoring devices (*n* = 37), visual observations (*n* = 31), camera traps (*n* = 10), proximity sensors (*n* = 5), animal‐borne cameras (*n* = 3), and genetics (*n* = 3), with 19 studies employing multiple techniques. Sample size reporting from observational studies varied and ranged broadly from 59 to 13,743 (median = 1132, SD = 4132) reported observations. Sample sizes from GPS or VHF studies ranged from 4 to 173 (median = 32, SD = 40) monitored deer. The thresholds at which intraspecific associations were considered corresponded to the methods employed. Observers' visual sightlines generally constituted associations in studies using visual observation; distances from 10 to 100 m (media*n* = 25 m, SD = 40 m) were used in GPS monitoring studies. Seasonal study periods were specific to questions of interest, but winter, parturition, summer, and the deer breeding period (hereafter, rut) were often considered as unique ecological time periods.

**FIGURE 2:**
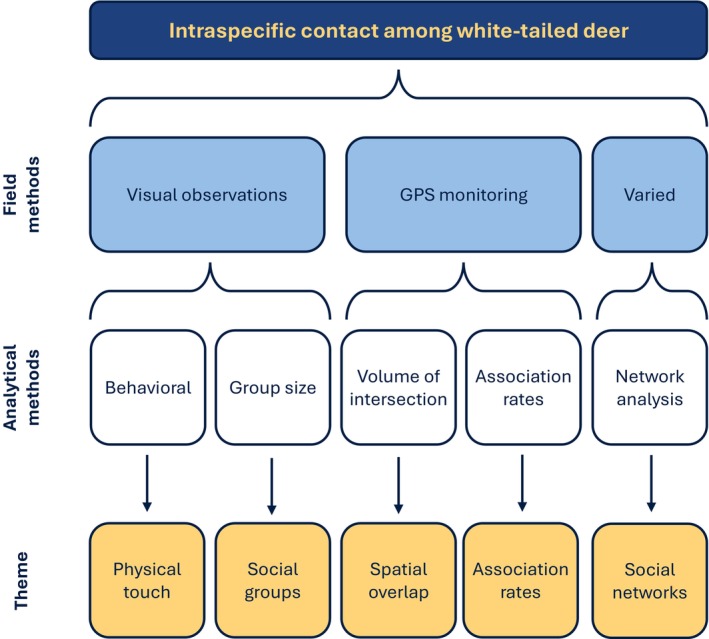
Conceptual diagram describing themes associated with intraspecific contact among white‐tailed deer ( *Odocoileus virginianus* ). Field and analytical methods indicate the primary approaches applied and are not inclusive of all prior or potential approaches.

During the literature review process, we identified five recurrent themes subdivided by the field and analytical methods applied in each study: physical touch (*n* = 19 studies), social groups (*n* = 25), spatial overlap (*n* = 20), association rates (*n* = 21), and social networks (*n* = 10) (Table 1; Figure 2). In the ensuing sections, we discuss intraspecific deer contact within these thematic contexts. Specifically, we synthesize key findings identified across themes relevant to disease transmission and discuss commonly applied field and analytical methods and their limitations. We considered 25 studies (36%) relevant to multiple themes, and aspects of deer ecology were often recurrent across themes.

**TABLE 1:** Summary of white‐tailed deer ( *Odocoileus virginianus* ) intraspecific contact literature grouped by research theme, field method, season, and location. Seasons were categorized corresponding to the reference authors' definitions. Field methods were categorized by the principal method used for data collection.

Research theme	Field method	Seasons^a^	Location	References^b^
Physical touch	Animal‐borne cameras	Rut	Missouri	Moll et al. (2009)
			Texas	Lavelle et al. (2012), Lavelle et al. (2014)
	Camera traps	Winter	Michigan	Courtney (2023)
			Wisconsin	Thompson et al. (2008)
		Year round	Maryland	Bai et al. (2024)
			Michigan	Vercauteren et al. (2007)
			Mississippi	Huang et al. (2025)
	VHF/GPS monitoring	Rut	Pennsylvania	Buderman et al. (2021)
	Visual observations	Parturition	Virginia	Schwede et al. (1993)
		Rut	Maryland	Shaw et al. (2006)
		Winter	Michigan	Ozoga (1972)
			Washington	Suring (1974)
		Year round	Georgia	LaGory (1986)
			Indiana	LaGory et al. (1981)
			Ohio	LaGory et al. (1981)
			Maryland	Rosenberry (1997)
			Michigan	Hirth (1977)
			Texas	Michael (1968), Hirth (1977), Hirth (1985)
Social groups	Camera traps	Winter	Indiana	Delisle et al. (2023)
			Wisconsin	Thompson et al. (2008)
	Visual observations	Parturition	Minnesota	Monteith et al. (2007)
			Virginia	Schwede et al. (1993)
		Rut	Minnesota	LaRue et al. (2007)
		Winter	Michigan	Courtney (2023)
		Year round	Alberta	Lingle (2003)
			Florida	Hardin et al. (1976)
			Georgia	LaGory (1986)
			Illinois	Hawkins and Klimstra (1970), Nixon et al. (1991), Nixon et al. (1994), Nixon and Mankin (2007), Nixon et al. (2010), Earegood‐McCarty (2019)
			Maryland	Rosenberry (1997)
			Mexico	Mandujano and Gallina (1996)
			Michigan	Hirth (1977)
			New York	Behrend (1966), Aycrigg and Porter (1997)
			Ohio	Sorensen and Taylor (1995)
			Pennsylvania	Stainbrook (2011)
			Texas	Hirth (1977), Kie and Bowyer (1999), Richardson and Weckerly (2007)
			Washington	Suring (1974), Gavin et al. (1984)
Spatial overlap	VHF/GPS monitoring	Parturition	Delaware	Haus et al. (2023)
			Virginia	Schwede et al. (1993)
		Rut	Pennsylvania	Buderman et al. (2021)
		Winter	Alberta	Habib et al. (2011)
			Illinois	Rustand (2010), Tosa et al. (2015), Tosa et al. (2017)
			New Jersey	Williams et al. (2008)
			Oklahoma	Long et al. (2015)
		Year round	Illinois	Schauber et al. (2007), Kjær et al. (2008), Schauber et al. (2015)
			Maryland	Roden‐Reynolds et al. (2022)
			Missouri	Walter, Beringer, et al. (2011)
			Nebraska	Walter, Baasch, et al. (2011)
			New York	Mathews and Porter (1993), Aycrigg and Porter (1997)
			South Carolina	Comer et al. (2005)
			Tennessee	Vargas Soto et al. (2025)
			South Dakota	Schuler (2006)
			Wisconsin	Magle et al. (2013)
Association rates	Animal‐borne cameras	Winter	Missouri	Moll et al. (2009)
	Proximity sensors	Summer	Pennsylvania	Gingery (2018)
		Rut	Texas	Lavelle et al. (2014)
		Winter	Illinois	Tosa et al. (2015), Tosa et al. (2017)
		Year round	Michigan	Lavelle et al. (2016)
	VHF/GPS monitoring	Parturition	Oklahoma	Long et al. (2022)
			Virginia	Schwede et al. (1993)
		Summer	Oklahoma	Long et al. (2014)
			Virginia	Schwede et al. (1994)
		Rut	Pennsylvania	Buderman et al. (2021)
			Texas	Lavelle et al. (2014)
		Winter	Alberta	Habib et al. (2011)
			Illinois	Rustand (2010), Tosa et al. (2015)
		Year round	Illinois	Schauber et al. (2007), Kjær et al. (2008), Schauber et al. (2015), Egan (2024), Egan et al. (2024)
			New York	Williams et al. (2014)
Social networks	Animal‐borne cameras	Rut	Texas	Lavelle et al. (2014)
	Camera traps	Rut	Mississippi	Hearst et al. (2021), Hearst et al. (2023)
			Nebraska	Egan et al. (2023), Egan (2024)
	Proximity sensors	Year round	Michigan	Wilber et al. (2019)
	VHF/GPS monitoring	Parturition	Oklahoma	Long et al. (2022)
		Year round	Illinois	Tosa et al. (2015), Koen et al. (2017)
	Visual observations	Year round	Illinois	Earegood‐McCarty (2019)

^a^ Broadly, rut was the fall breeding period, winter was the period between rut and parturition, parturition was the early summer period corresponding to birth, and summer was the period between parturition and the onset of rut. Year‐round studies encompassed multiple seasonal study periods.

^b^ These studies considered indirect transmission: (Schauber et al. 2007; Rustand 2010; Wilber et al. 2019; Hearst et al. 2021, 2023; Egan et al. 2023; Vargas Soto et al. 2025).

### 2.2 Physical Touch

Several deer behaviors result in direct physical contact (i.e., touching), a primary concern for disease transmission. Mutual behaviors, such as nose‐touching and grooming (Lavelle et al. 2012), were considered high risk for disease transmission while agonistic behaviors, such as strikes, rushes, and flails (Ozoga 1972; Hirth 1977), were considered lower risk (Thompson et al. 2008). High‐risk mutual behaviors were most frequently exhibited between females, their offspring, and other conspecifics (LaGory et al. 1981; Moll et al. 2009). Rut was inclusive of high disease risk behaviors, including tending (i.e., male defense of females, often including males licking females' tarsal glands and urogenital regions to assess female readiness to breed [DeYoung and Miller 2011]), copulation, and agonistic dominance interactions (i.e., sparring) between males (Lavelle et al. 2014).

Deer behavior is well‐documented (DeYoung and Miller 2011), including quantifying the frequency of physical touch. Studies reporting rates of physical touch, however, documented much variation: 0.05–0.32 (LaGory 1986), 0.00–0.92 (Suring 1974), and 1.22–1.60 (Ozoga 1972) touches per hour. Similar discrepancies were reported for rates of all social interactions, not just those resulting in physical touch. Low rates of 0.05–0.22 social interactions per hour (LaGory 1986; Schwede et al. 1993) and 0.00–0.34 social interactions per hour (Courtney 2023; Huang et al. 2025), as well as relatively higher rates of 2–4 (Ozoga 1972), 0.0–5.2 (Hirth 1977, 1985), 0.0–5.4 (Suring 1974), and 0–11 (Rosenberry 1997; Shaw et al. 2006) interactions per hour were reported. The duration and corresponding disease risk of these interactions were reported infrequently. Nursing events averaged 4.3 min (max = 9.5 min) (Hirth 1985), and a single mouth‐to‐body grooming event lasted 7 min (Moll et al. 2009). Yearling male sparring events of 11 and 19 min were also observed (Rosenberry 1997).

The variability of estimated rates of physical touch likely corresponds to differences in deer behavior among demographic classes, habitats, and time periods. Adult females and their offspring touched much more frequently than other sex–age pairs (Hirth 1977). Social interactions were more frequent at bait sites than at other food sources (Courtney 2023; Huang et al. 2025). More specifically, agonistic behaviors were more frequent in pasture habitats, and mutual behaviors were more frequent in forested habitats (LaGory 1986). Agonistic behavior frequency did not, however, vary between natural and baited feeding sites (Thompson et al. 2008). Temporally, agonistic behaviors involving females peaked during summer after parturition (Michael 1968; Schwede et al. 1993), and agonistic behaviors involving males peaked during rut (Michael 1968; Rosenberry 1997). Comparatively, agonistic behaviors at anthropogenic bait sites peaked in winter (January–March) and were lowest during rut (October–December) (Bai et al. 2024).

Field methods for assessing physical touch included active visual observations, camera traps, animal‐borne cameras, and GPS monitoring (Table 1) with behaviors generally quantified as counts, analyzed using descriptive summary statistics. Studies only using visual observations (Michael 1968; Ozoga 1972; Suring 1974; Hirth 1977) dissipated as improved technologies allowed for greater data volume using newer methods. Application of animal‐borne cameras produced relatively low intraspecific contact data volume (Moll et al. 2009; Lavelle et al. 2012, 2014), limiting their utility. Quantifying behavioral states using GPS monitoring also met with limited success, potentially due to the sampling rate (Buderman et al. 2021). Applications of camera traps to assess deer behavior included identification of social interactions at anthropogenic food sources (Thompson et al. 2008; Courtney 2023; Bai et al. 2024; Huang et al. 2025) and deer breeding facilities (Vercauteren et al. 2007; Khouri et al. 2022); however, these studies provide limited insights into disease transmission dynamics because they did not identify individuals.

### 2.3 Social Groups

The foundation of the deer social group structure is the matriarchal family group. Adult females gave birth in May and June, and during this period, they isolated from previous‐year offspring (Monteith et al. 2007; Nixon and Mankin 2007; Schauber et al. 2015). Recorded female group sizes were correspondingly lowest in June, averaging 1.4–3.0 deer per group (Nixon et al. 1991; Schwede et al. 1993; Kie and Bowyer 1999; Richardson and Weckerly 2007). Thereafter, yearling females began rejoining their mothers, forming progressively larger social groups throughout summer (Schwede et al. 1993; Richardson and Weckerly 2007; Nixon et al. 2010). Likely corresponding to this social isolation and subsequent group reformation, a broad range of females (31%–82%) were observed alone across studies during summer (Behrend 1966; Nixon and Mankin 2007; Earegood‐McCarty 2019). Matriarchal family groups, inclusive of an eldest female, her generational offspring, and sometimes other related individuals (e.g., half‐siblings), reached their largest sizes in winter, averaging 5–8 deer per group (Nixon et al. 1991, 2010; Schwede et al. 1993; Lingle 2003; Richardson and Weckerly 2007; Courtney 2023) with 58%–99% of females observed in such groups (Behrend 1966; Nixon and Mankin 2007; Earegood‐McCarty 2019). Matriarchal groups began disbanding in late winter prior to parturition, and the cycle repeated (Schwede et al. 1993; Richardson and Weckerly 2007). Similar patterns were documented among subspecies, though group sizes were generally smaller (Hardin et al. 1976; Gavin et al. 1984; Mandujano and Gallina 1996), which likely corresponds to less suitable habitat on the edge of the species' range (Brown 1984).

Male yearlings began dispersing when their mothers isolated themselves for parturition; thereafter, 11%–30% of male yearlings remained alongside natal matriarchal females until they dispersed prior to or during rut (Nixon et al. 1994; Rosenberry 1997; Nixon and Mankin 2007). After dispersal, adult males (i.e., those > 1.5 years of age) rarely (< 1%) associated with their natal matriarchal groups (Nixon et al. 1994; Nixon and Mankin 2007) and more frequently associated with yearling males (Rosenberry 1997). Post‐dispersal males formed bachelor groups with unrelated males (Nixon and Mankin 2007). These bachelor groups sometimes disbanded during the rut (Hawkins and Klimstra 1970), but were generally stable year‐round, averaging two deer per group (Nixon et al. 1991; Richardson and Weckerly 2007) with 18%–54% of adult males observed alone (Nixon et al. 1994; Nixon and Mankin 2007).

Mixed‐sex social groups composed 2%–40% of observed groups, which resulted in increased estimates for group sizes (Hirth 1977; Nixon et al. 1994; Kie and Bowyer 1999; Lingle 2003; Richardson and Weckerly 2007; Courtney 2023) dependent on whether yearlings were considered members of or separate from matriarchal family groups (Monteith et al. 2007). The largest groups were observed during winter (Hirth 1977; Sorensen and Taylor 1995; Kie and Bowyer 1999; Lingle 2003; Richardson and Weckerly 2007), except among the Sinaloa subspecies (Mandujano and Gallina 1996). Corresponding to habitat, landscape characteristics influenced group size dynamics. The largest congregations were observed in open habitats, such as grasslands and agricultural fields, while forested and anthropogenic residential habitats generally contained smaller groups (Hirth 1977; LaGory 1986; Courtney 2023; Delisle et al. 2023). These observations were consistent with sightings of winter yarding groups, which resulted in groups of > 50 deer (Hirth 1977; Courtney 2023) with groups as large as 200 reported (Ozoga 1972). Anthropogenic food plots have shown greater potential for indirect disease transmission than natural feeding areas (Huang et al. 2025), and the resultant mixed‐sex yarding groups likely present high potential for between‐group transmission, warranting consideration of management options to limit spread in diseased areas.

The primary methodology for assessing group sizes was visual observations (Table 1). The principal means were road‐based surveys wherein the observer traveled slowly along established roadways (LaRue et al. 2007; Richardson and Weckerly 2007; Stainbrook 2011; Courtney 2023) and use of blinds or other concealment in pre‐selected locations (Suring 1974; Hirth 1977; LaGory 1986; Rosenberry 1997). Several studies additionally employed ear tags (Hawkins and Klimstra 1970; Nixon et al. 1991; Rosenberry 1997; Earegood‐McCarty 2019) or radio telemetry (Schwede et al. 1993; Nixon et al. 1994; Aycrigg and Porter 1997; Rosenberry 1997) to improve the repeat identification of individuals and their social groups. Camera traps were also used to assess group sizes but reported relatively small groups (1.12–1.32 [Thompson et al. 2008] and 2.31–3.20 [Delisle et al. 2023]) unless detectability was accounted for (Delisle et al. 2023).

### 2.4 Spatial Overlap

Herein, we defined spatial overlap as co‐occurrence at the home range scale (i.e., home range overlap). Early estimation of spatial overlap assumed deer with greater spatial overlap had greater contact rates and therefore greater intra‐ rather than inter‐group transmission rates (Walter, Baasch, et al. 2011; Walter, Beringer, et al. 2011). Estimates of direct and indirect association rates (Schauber et al. 2007; Rustand 2010; Long et al. 2015) and similar movements (Vargas Soto et al. 2025) were later correlated to spatial overlap, providing validation for the initial assumptions underlying this approach and its feasibility as a proxy when higher resolution data are unavailable. Spatial overlap was influenced by anthropogenic landscape characteristics, with urban deer exhibiting greater overlap, likely corresponding to limited habitat availability (Walter, Beringer, et al. 2011). Baiting in a suburban landscape exacerbated this transmission risk by decreasing distances between collared individuals (Roden‐Reynolds et al. 2022).

Deer social group dynamics suggest females in close proximity are typically members of the same matriarchal family groups. Analyses of spatial‐genetic relatedness confirmed this conclusion, positively correlating increasing genetic relatedness among females to spatial overlap (Mathews and Porter 1993; Comer et al. 2005; Magle et al. 2013). In this context, spatial overlap has been used as an alternative metric for defining social groups in lieu of genetics or visual observations (Tosa et al. 2015, 2017; Haus et al. 2023). Subsequent analyses compared association rates within and between social groups as described in the next section.

Though estimation of spatial overlap can be completed using camera traps (Kumbhojkar et al. 2020), efforts among deer were limited to the more common application of VHF and GPS monitoring (Table 1). Estimates of spatial overlap among deer initially used minimum convex polygons (MCP) to determine seasonal deer home ranges and visually identify corresponding social groups (Mathews and Porter 1993; Schwede et al. 1993; Aycrigg and Porter 1997). Williams et al. (2008) used Chamberlain and Leopold (2002)'s overlap index to improve MCP assessments. However, the volume of intersection (VI) has become the most used technique for estimating deer spatial overlap (Walter, Baasch, et al. 2011; Magle et al. 2013; Long et al. 2015; Tosa et al. 2017). The VI for a deer dyad (i.e., a pair of deer) is calculated by estimating utilization distributions (UD) using kernel density estimation (KDE) for each deer and then calculating the three‐dimensional intersection, thereby improving estimation in comparison to MCP overlap (Seidel 1992; Millspaugh et al. 2004). Joint potential path area (Long et al. 2015) and joint utilization distribution (Kjær et al. 2008) have also been considered as alternatives to VI, but these approaches have not been widely applied (Joo et al. 2018). Recently, deer home ranges have been estimated using Brownian bridge movement models (BBMM) with spatial overlap calculated as the two‐dimensional intersection of resultant polygons (Buderman et al. 2021; Wehr et al. 2024); however, a comparative assessment of the VI and BBMM intersection has not been conducted.

A supporting approach to estimating spatial overlap is estimating similarities in movement. Adapted from Shirabe (2006), Long and Nelson (2013) estimated the dynamic interaction index (DI) as a metric assessing temporal similarities in step lengths and turning angles. DI and VI were positively correlated among deer (Long et al. 2014; Tosa et al. 2015; Haus et al. 2023), with DI > 0.2 used to identify social groups (Tosa et al. 2015, 2017). Another approach was developed by Schauber et al. (2007) determining similarity in movements using Pearson's correlation coefficient (*r*) to estimate the temporal correlation of *Z* for each dyad, where *Z* is the sum of the *x* and *y* coordinates at each time step (i.e., *Z* = *X* + *Y*). This metric has been used to delineate social groups (*r* > 0.45) (Schauber et al. 2015; Gilbertson et al. 2025), but we warrant caution in its application. This metric has the potential to produce misleading results because deer located northwest or southeast of one another could be considered spatially proximate when at disparate locations. For example, given that *X*_*i*_ = 0, *Y*_*i*_ = 100, *X*_*j*_ = 100, and *Y*_*j*_ = 0, locations would be considered near one another (*Z*_*i*_ = 100, *Z*_*j*_ = 100) though individuals are not in the same location. More recently, Vargas Soto et al. (2025) used a “messy follow‐the‐leader” approach to assess the probability of two deer occupying the same patches in subsequent time steps, which they correlated to an increased probability of disease transmission.

### 2.5 Association Rates

Estimates of association rates corresponded to social group dynamics. Using 50% VI to delineate social groups, Schauber et al. (2007) reported within‐group association rates of 5%–25% at a 10‐m threshold and 25%–85% at a 100‐m threshold with 1–2‐h relocation intervals. Using MCP overlap to define social groups, Long et al. (2014) reported a between‐group association rate of 2%, a within‐group association rate of 7%, and a within‐group association rate between two males confirmed as members of the same bachelor group of 76% using a 50‐m threshold and 15–30‐min relocation intervals. Association rates of confirmed mother–offspring dyads were high immediately following parturition (> 16% during the first week and > 67% by month three using a 130‐m threshold and 10‐min relocation intervals [Gingery 2018]), and remained high thereafter (> 60% using a 20‐m threshold via intermittent telemetry‐assisted visual observations [Schwede et al. 1993; Schwede et al. 1994]). Comparatively, between‐group association rates were consistently low (< 3%) at distance thresholds < 50 m using relocation intervals of 15 min–6 h (Schauber et al. 2007; Kjær et al. 2008; Habib et al. 2011; Lavelle et al. 2014; Tosa et al. 2017), though relatively higher (< 20%) at a greater distance threshold (100 m, 2‐h relocation intervals [Schauber et al. 2007]). Seasonally, association rates were higher during winter than summer (Kjær et al. 2008; Williams et al. 2014), potentially corresponding to female isolation during parturition (Schwede et al. 1993). Association rates also corresponded to the lunar cycle, being greater during full moons (Kjær et al. 2008). Indirect association rates were also assessed using GPS monitoring; in these studies, spatially concurrent locations (< 100 m) within 30‐day temporal thresholds were considered indirect associations (Schauber et al. 2007; Rustand 2010).

Attempts to estimate duration of association events added limited depth to understanding disease‐transmission potential. Tosa et al. (2017) used proximity loggers to determine the duration deer were within 2 m, finding very short periods for both adults and juveniles (< 1–10 s). Buderman et al. (2021) estimated duration of mating events using number of consecutive locations (2‐h relocation interval) wherein a male and female were < 100 m apart at 7.9 h (range = 1–78.9 h). Predictions of landscape characteristics corresponding to higher association probabilities were similarly limited, as the applicability of these models to alternative sites was restricted (Egan 2024; Egan et al. 2024).

Association rate estimation among deer was primarily conducted using GPS monitoring devices or proximity sensors (Table 1), which required temporal and spatial thresholds within which to consider contacts. Temporal thresholds were typically determined by relocation intervals (Long et al. 2014, 2022), but have been shown to correlate with estimated association rates, with longer intervals underestimating true values (Yang et al. 2023). Comparatively, distance thresholds were specific to study objectives and varied widely (< 1–100 m). Analytical approaches for establishing study‐specific threshold distances included identification of natural breaks in pairwise distances between monitored individuals using local minima (Long et al. 2014, 2022) and identification of the distance maximizing association rates within a user‐specified range (Kaur et al. 2024).

Several metrics were proposed to assess association rates (Long et al. 2014). The simplest approach was proximity analysis, which calculates the proportion of spatially concurrent locations for each dyad (Bertrand et al. 1996; Long et al. 2014). A similar alternative was Cole's coefficient of association, calculated as twice the number of spatially concurrent locations divided by the total number of locations recorded for each dyad member (Cole 1949; Long et al. 2014). Other metrics included those reliant on the distance between dyad members (Doncaster 1990; Kenward et al. 1993; Benhamou et al. 2014) or spatial overlap of dyad members (Minta 1992; Atwood and Weeks Jr. 2003; Millspaugh et al. 2004). Most of these metrics can be estimated with functions from the *wildlifeDI* R package, and comparisons of these metrics for assessing deer association rates returned similar conclusions (Long et al. 2014, 2022; Long 2024).

Interpretation of association rates is most effective when intra‐ and inter‐group dynamics are delineated. Some studies, however, did not address this issue (Moll et al. 2009; Lavelle et al. 2014). Attempts to address this distinction included assessments of mother–offspring dyads (Schwede et al. 1994), social group status using visual observations (Long et al. 2014), and application of spatial metrics (Schauber et al. 2015; Tosa et al. 2015). The implementation of spatial metrics was most universally applicable because it can be performed using GPS monitoring data without supporting visual observations. Studies using these metrics on a continuous scale included the application of the greatest distance between dyad locations (Williams et al. 2014) and VI (Habib et al. 2011) as predictors of association rates. Comparatively, several assessments used threshold values to delineate within‐ versus between‐group associations (Long et al. 2014). The most widely used threshold was VI, with dyads of high VI (> 40%–50%) considered members of the same social group (Schauber et al. 2007, 2015; Tosa et al. 2015). Correlated with VI, DI was another useful alternative (Long et al. 2014; Tosa et al. 2015).

### 2.6 Social Networks

Social network analyses of deer produced several ecologically relevant outcomes for understanding disease transmission. Comparisons of social network metrics across habitats with differing levels of habitat connectivity, for example, indicated greater social connectivity among deer living in more fragmented landscapes (Koen et al. 2017). Fencing to prevent deer access to livestock food sources may combat indirect disease spread, as social network connectivity, including interspecies connectivity, decreased when fencing was implemented (Wilber et al. 2019). Identification of super‐spreaders (i.e., individuals that contact many others) also presented potential management implications (Lavelle et al. 2014) as the development of male social hierarchies indicated disproportionately large influences of socially dominant bucks on indirect transmission (Hearst et al. 2021, 2023; Egan et al. 2023). Removal of individual bucks from these models significantly decreased social connectivity, supporting the use of hunter harvest to decrease intraspecific contact (Egan et al. 2023; Hearst et al. 2023).

Field methods supporting assessments of social networks varied widely (Table 1). The most insightful method was the application of camera traps to identify indirect contact at scrapes (i.e., scent‐marking sites used during rut with acknowledged potential for disease transmission [Kinsell 2010; Huang, Demarais, Banda, et al. 2024]) by identifying individual males (Hearst et al. 2021, 2023; Egan et al. 2023). Subsequent analyses were used to assess the interconnectivity of study populations and identify potential disease transmission pathways. Social network diagrams were useful for visualizing connections between individuals or groups. These diagrams represented individuals as a network of “nodes” with “edges” as lines interconnecting the nodes; often, edges were weighted by the association rate between nodes. Typical metrics included network‐level metrics—mean path length (i.e., average number of connections needed to connect two individuals) and edge density (average number of observed connections divided by number of possible connections)—and individual‐ or node‐level metrics—closeness (i.e., connections needed to reach all other individuals), betweenness (i.e., frequency of an individual occurring on shortest path between other individuals), and centrality (i.e., inclusive of closeness and betweenness) (Egan et al. 2023; Hearst et al. 2023). These scores were often used in tandem and collectively represent intraspecific contact rates, with higher values typically corresponding to greater social interconnectivity (Silk et al. 2017; Egan et al. 2023; Hearst et al. 2023). Similar to association rates, these metrics were highly sensitive to the distance at which two individuals were considered associated (Tosa et al. 2015). Social network analyses were generally conducted at small spatial extents when seeking to understand within‐group transmission (Hearst et al. 2021; Egan et al. 2023) and at larger scales for between‐group transmission (Koen et al. 2017; Earegood‐McCarty 2019), though Hearst et al. (2023) considered both simultaneously.

Likely corresponding to the interdisciplinary applicability of social network analyses, several software applications supported their use. Gephi (Bastian et al. 2009; Hearst et al. 2021), Pajek (Lavelle et al. 2014; Mrvar and Batagelj 2016), and Ucinet (Borgatti et al. 2002; Tosa et al. 2015) were standalone programs used for deer. R packages, including *sna* (Tosa et al. 2015; Butts 2024), *tnet* (Opsahl 2009; Koen et al. 2017), *EpiModel* (Jenness et al. 2018; Earegood‐McCarty 2019), and *wildlifeDI* (Long et al. 2014, 2022), were also used, with the *igraph* (Csardi and Nepusz 2006; Earegood‐McCarty 2019; Csardi et al. 2024) and *GGally* (Long et al. 2022; Schloerke et al. 2024) packages used for visualization.

### 2.7 Synthesis

Seasonal dynamics of deer behavior and ecology are well understood (e.g., parturition, rut, and dispersal), and our literature review supported the prevailing understanding of these dynamics (DeYoung and Miller 2011). Matriarchal family groups are generally composed of mothers, their female offspring, and pre‐dispersal juvenile males, while post‐dispersal adult males form small bachelor groups (Monteith et al. 2007; DeYoung and Miller 2011). These social dynamics can vary seasonally; for example, matriarchal family groups disband during parturition and reform throughout summer (Nixon and Mankin 2007). Visual observations of social groups indicated physical touching was much greater among mother–offspring dyads (Hirth 1977), with high disease risk male–female interactions occurring primarily during rut (Lavelle et al. 2014).

Corresponding assessments of potential disease transmission events using GPS monitoring supported these visual observations. Specifically, association rates of mother–offspring dyads were high during the first several months of life (Schwede et al. 1993, 1994; Gingery 2018). Within‐group association rates were also generally much higher than between‐group association rates (Schauber et al. 2007). However, despite a strong understanding of behavioral dynamics among deer and the increasing application of GPS monitoring and disease surveillance data, we did not identify any studies of deer that comprehensively assessed seasonal contact rates among demographic classes. Further, we identified no studies in which disease status was considered in these interactions. We address these knowledge gaps in the following case study.

## 3 Case Study

### 3.1 Background

We developed this case study to quantify contact rates (using association rates as a proxy) of deer year‐round spanning a suite of sex‐age class dyads. Prior work has not provided a comprehensive summary of season‐ and demographic‐specific contact rates, and we sought to fill this gap. Further, we incorporated post‐mortem assessments of CWD to address a second knowledge gap, comparing contact rates among infected and uninfected individuals. Estimation of contact rates rarely considers this comparison, and more specifically, there are few studies comparing deer movements between infected and uninfected individuals. Addressing these knowledge gaps was important for informing disease modeling efforts because such efforts require contact rates for estimation of disease transmission (McCallum et al. 2001; White et al. 2017).

### 3.2 Data Collection

In 2012, the Pennsylvania Game Commission recorded the state's first positive detections of CWD in free‐ranging deer, which prompted the establishment of Disease Management Area 2 in southcentral Pennsylvania, United States (PGC 2020). The CWD established zone (Figure 1B) represents the core of this area including the locations of the first positive CWD detections. To support CWD management objectives, 194 deer in the CWD established zone were monitored using GPS collars (Vertex Light, Vectronic Aerospace, Berlin, Germany) during February 2018–October 2020 and February 2023–December 2024. Locations were recorded every 30 min, with mortality signals triggered after 8 h of inactivity. To promote CWD testing, a reward was allocated to hunters reporting the harvest of monitored deer. Sex (male or female) and age (adult [> 12 months] or juvenile [< 12 months]) were assessed during captures using physical characteristics. All capture and handling protocols were approved by The Pennsylvania State University institutional animal care and use committee (IACUC protocols # 201800026 and # 202202225). Postmortem CWD testing (positive or undetected) used immunohistochemistry or enzyme‐linked immunosorbent assays of the obex and retropharyngeal lymph nodes. We assumed CWD‐positive deer were infected for the entire monitoring period as the monitoring durations (median = 288 days, SD = 160, range = 18–793) were generally shorter than typical post‐infection survival (693 ± 27–956 ± 107 days [Johnson et al. 2011]).

### 3.3 Data Curation and Analysis

Our analysis incorporated thematic concepts of intraspecific contact identified by our literature review, typically assessed using GPS monitoring data: spatial overlap and association rates (Table 1; Figure 2). We considered three social‐ecological time periods: winter (21 December–10 May), summer (11 May–15 October), and rut (16 October–20 December). These time periods were determined by prior analyses in Pennsylvania (Gingery 2018; Buderman et al. 2021). We did not consider parturition separately from summer to encapsulate the entire period of female isolation and matriarchal group reformation. We collated results by age class (juveniles < approximately 18 months old, adults > approximately 18 months old), sex (female, male), and CWD status (positive, undetected). If an individual captured as a juvenile survived to become an adult, we reclassified its status at the onset of winter. Prior to our analyses, we censored 14 deer whose relocation intervals were inconsistent, resulting in a temporal mismatch between monitored individuals. Monitoring of four individuals captured during winter 2018 ceased during the winter 2019 capture period due to mortality or collar failure; we censored locations recorded by these four deer after 21 December 2018 due to the resultant temporal mismatch between their recorded locations and locations recorded by deer whose monitoring periods began late in winter 2019. We excluded deer with < 372 recorded locations (i.e., 2 weeks of monitoring) during specific seasons.

We conducted all data curation and analyses in R (R Core Team 2025) using code published by Wehr and Walter (2025). We calculated season‐specific association rates using the *prox* function in the *wildlifeDI* package using a 50‐m distance threshold (a middling value among prior studies) and a 5‐min time threshold (Long et al. 2014, 2022; Long 2024). The output was the proportion of locations in which dyad members recorded spatial–temporal concurrent locations within 50 m within 5 min of one another (i.e., count of concurrent locations/count of possible concurrent locations). We assessed movement correlation using DI calculated for each dyad using the *DI* function in the *wildlifeDI* package with a 5‐min time threshold (Long et al. 2014, 2022; Long 2024). DI is representative of correlation in step lengths and turning angles (Long and Nelson 2013). We calculated season‐specific spatial overlap using VI (Millspaugh et al. 2004). We used the *amt* package to calculate VI by first producing KDE UDs for each deer during each season using the *hr_kde* function at the 95% home range level and then using the *hr_overlap* function to calculate VI (Signer et al. 2019; Signer and Fieberg 2024). We censored all dyads with 0% VI because we considered contact between these deer unlikely.

**FIGURE 3:**
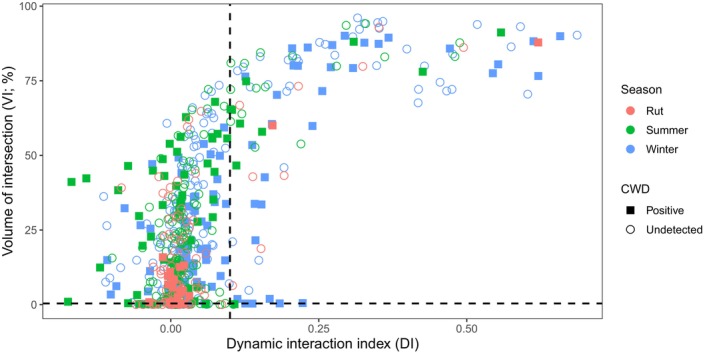
Spatial correlation of white‐tailed deer ( *Odocoileus virginianus* ) in southcentral Pennsylvania, United States, 2018–2020 and 2023–2024. Volume of intersection (VI) represents the spatial overlap of deer dyads' home ranges. The dynamic interaction index (DI) represents correlation in step lengths and turning angles of dyads. The horizontal and vertical dashed lines represent the thresholds (VI > 40%; DI > 0.15) used to assess deer social group status. Rut was from 16 October to 20 December; summer was from 11 May to 15 October; winter was from 21 December to 10 May. Chronic wasting disease (CWD) was assessed post‐mortem for all available individuals.

We presented the results of these calculations in the context of social groups. We initially considered individuals with VI > 40% members of the same social group (Schauber et al. 2007, 2015; Tosa et al. 2015). We also considered an additional threshold wherein deer with DI > 0.15 were considered members of the same social group. This threshold was lower than previously applied but represented a more natural transition in our data (Figure 3) in the context of prior studies (Tosa et al. 2015, 2017).

We fit four generalized linear mixed‐effect models (GLMMs) using the *glmmTMB* package (Brooks et al. 2017). The first three models used all available dyads. Model 1 used VI as the response and DI as the predictor. Models 2 and 3 used the association rate as the response variable with VI and DI as the predictor variables, respectively. All three of these models were binomial logit‐linked GLMMs fit using dyad ID as a random effect. We were unable to consider other covariates as predictors of association rates because we identified an insufficient number of associations to address the inherently interactive effects of sex, age class, and season. For our fourth model, we subset our data to include only dyads clearly descriptive of matriarchal family groups. We defined these groups as having VI > 40% and DI > 0.15, monitored during winter when relationships are most stable, and sex‐age combinations inclusive of adult females interacting with other adult females, juvenile females, or juvenile males, and juvenile females interacting with other juvenile females.

### 3.4 Results

**FIGURE 4:**
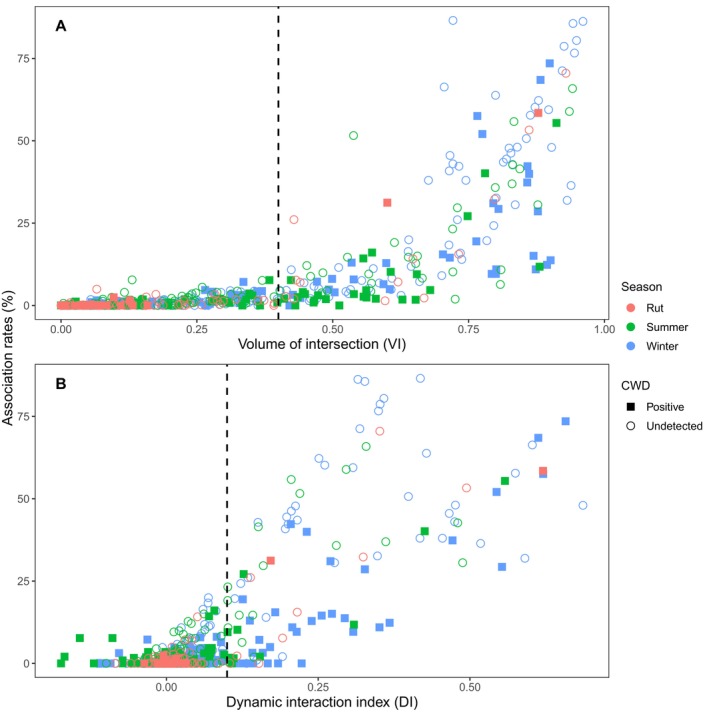
Spatial correlation of white‐tailed deer ( *Odocoileus virginianus* ) in southcentral Pennsylvania, United States, 2018–2020 and 2023–2024. Association rates represent the proportion of concurrent locations for a deer dyad. Volume of intersection (VI; Panel A) represents the spatial overlap of deer dyads' home ranges. The dynamic interaction index (DI; Panel B) represents correlation in step lengths and turning angles of deer dyads. The vertical dashed lines represent the thresholds (VI > 40%; DI > 0.15) used to assess deer social group status. Rut was from 16 October to 20 December; summer was from 11 May to 15 October; winter was from 21 December to 10 May. Chronic wasting disease (CWD) was assessed post‐mortem for all available individuals.

We analyzed 1,899,800 locations recorded among 180 monitored deer (median = 10,057, SD = 8164, range = 445–31,402) in southcentral Pennsylvania during 2018–2020 and 2023–2024. Of the analyzed deer, 37 tested positive for CWD post‐mortem. We identified 592 season‐specific deer dyads with > 0% VI, including dyads monitored during multiple seasons. Among these seasonal dyads, association rates, VI, and DI were all strongly correlated (Table 2; Figures 3 and 4). Further, 169 seasonal dyads had high spatial overlap (VI > 40%). Among high VI dyads, 76 also had high movement correlation (DI > 0.15). Association rates of high VI dyads (median = 12.7%, SD = 22.8%, range = 0.0%–86.6%) appeared greater than those of low VI dyads (median = 0.1%, SD = 1.3%, range = 0.0%–7.7%; Table 3). Association rates of dyads with high VI and high DI appeared to have the greatest association rates (median = 41.9%, SD = 22.4%, range = 2.0%–86.6%).

**TABLE 2:** Assessments of interactions among white‐tailed deer ( *Odocoileus virginianus* ) in southcentral Pennsylvania, United States, 2018–2020 and 2023–2024. Association rates (AR), volume of intersection (VI), and dynamic interaction index (DI) were correlated among deer dyads.

Response	Predictor	*β*	SE	*z*	*p*	Figures
VI	DI	8.62	1.05	8.18	< 0.001	Figure 3
AR	VI	6.95	0.89	7.79	< 0.001	Figure 4A
AR	DI	8.58	1.00	8.57	< 0.001	Figure 4B

**TABLE 3:** Association rates of 592 season‐specific white‐tailed deer ( *Odocoileus virginianus* ) dyads in southcentral Pennsylvania, United States, 2018–2020 and 2023–2024, summarized by sex/age class and season. Association rates are reported as the median percentage (range) of global positioning system locations within 50 m and 5 min for each sex/age class combination during each period. Social group metrics used included the dynamic interaction index (DI) and volume of intersection (VI).

Sex/age class combinations^a^	Winter^b^	Summer^b^	Rut^b^	Social group metric
FA–FA	35.3 (2.1–86.6)	42.7 (30.6–55.4)	55.9 (53.3–58.5)	High movement correlation (DI > 0.15) AND High spatial overlap (VI > 40%)
FJ–FA	29.9 (9.6–62.2)	11.8 (2.0–36.9)	15.6 (7.7–32.3)	
FJ–FJ	48.1 (42.3–78.7)	65.9^c^	50.9 (31.2–70.5)	
FJ–MA	NA^d^	NA^d^	NA^d^	
MA–FA	NA^d^	NA^d^	NA^d^	
MA–MA	59.4^c^	34.9 (29.7–40.1)	NA^d^	
MJ–FA	40.4 (37.3–43.5)	NA^d^	NA^d^	
MJ–FJ	45.5 (40.9–86.3)	48.7 (41.5–55.8)	NA^d^	
MJ–MA	NA^d^	NA^d^	NA^d^	
MJ–MJ	75.7 (71.3–80.5)	58.9^c^	NA^d^	
FA–FA	15.9 (2.1–86.6)	4.5 (0.8–55.4)	53.3 (14.2–58.5)	High spatial overlap (VI > 40%)
FJ–FA	13.9 (0.0–62.2)	8.3 (0.0–36.9)	15.6 (7.7–32.3)	
FJ–FJ	46.3 (26.0–78.7)	46.5 (27.1–65.9)	31.2 (2.7–70.5)	
FJ–MA	7.2^c^	NA^d^	2.0^c^	
MA–FA	4.0 (2.7–9.1)	6.8^c^	1.8 (1.4–2.3)	
MA–MA	30.8 (2.1–59.4)	29.7 (0.0–40.1)	NA^d^	
MJ–FA	22.3 (5.1–43.5)	7.8 (4.7–10.8)	NA^d^	
MJ–FJ	42.8 (1.4–86.3)	16.4 (4.4–55.9)	7.1^c^	
MJ–MA	18.4 (7.9–19.7)	NA^d^	NA^d^	
MJ–MJ	17.7 (6.9–80.5)	34.6 (10.2–58.9)	16.5 (7.1–26.1)	
FA–FA	0.0 (0.0–4.2)	0.0 (0.0–5.5)	0.0 (0.0–2.5)	Low spatial overlap (VI < 40%)
FJ–FA	0.1 (0.0–4.5)	0.0 (0.0–7.7)	0.0 (0.0–0.1)	
FJ–FJ	1.1 (0.4–4.4)	1.2 (0.1–4.0)	1.1 (0.1–3.3)	
FJ–MA	0.2 (0.0–3.2)	0.3 (0.0–1.3)	0.0 (0.0–0.0)	
MA–FA	0.0 (0.0–4.4)	0.0 (0.0–1.8)	0.0 (0.0–1.5)	
MA–MA	1.2 (0.0–7.1)	0.1 (0.0–2.5)	0.1 (0.0–0.2)	
MJ–FA	0.0 (0.0–1.6)	0.3 (0.0–2.6)	0.0 (0.0–0.8)	
MJ–FJ	0.9 (0.0–3.4)	0.7 (0.0–4.4)	0.3 (0.0–4.9)	
MJ–MA	0.8 (0.0–4.3)	0.1 (0.0–7.6)	0.0 (0.0–0.7)	
MJ–MJ	0.9 (0.2–2.3)	0.4 (0.0–7.8)	0.0 (0.0–1.8)	

^a^ Sex/age class combinations represent each possible combination; A = adult, F = female, J = juvenile, and M = male.

^b^ Winter was from 21 December to 10 May; summer was from 11 May to 15 October; rut was from 16 October to 20 December.

^c^ Medians presented without ranges consisted of only one dyad.

^d^ Combinations without values (NA) indicate no dyads were documented meeting the social group metric thresholds.

Our assessment of association rates of deer in matriarchal family groups during winter (*n* = 43 dyads) compared dyads inclusive (*n* = 22) and exclusive (*n* = 21) of CWD‐infected deer. The results indicated that associated rates among dyads with CWD‐infected deer did not differ from those without infected deer (*β* = 0.92 ± 0.65, *z* = 1.42, *p* = 0.16). Further, when all dyads were included, association rates of high VI dyads inclusive of CWD‐infected deer (median = 9.6%, SD = 18.6%, range = 0.0%–73.5%) appeared similar to those of high VI dyads exclusive of CWD‐infected deer (median = 15.7%, SD = 24.1%, range = 1.4%–86.6%), and association rates of low VI dyads inclusive of CWD‐infected deer (median = 0.0%, SD = 1.4%, range = 0.0%–7.7%) appeared similar to low VI dyads in which CWD was not detected (median = 0.0%, SD = 1.4%, range = 0.0%–7.7%). We could not, however, assess these trends statistically.

### 3.5 Discussion

The available literature lacked a comprehensive assessment of contact rates among deer. We sought to address this knowledge gap, and our case study provides a thorough, though still imperfect, assessment (Table 3). Our results are likely explained by deer social dynamics. For example, parturient females isolate themselves during and immediately following parturition (Schwede et al. 1993; DeYoung and Miller 2011). Corresponding to this isolation, our results appear to indicate decreased association rates between adult females and potential members of their matriarchal social groups (i.e., adult females and juveniles of either sex) during summer in comparison to winter. Supporting assessments that bachelor groups were independent from matriarchal family groups (DeYoung and Miller 2011), our results suggest association rates among adult males were greatest with other adult males, while juvenile male association rates were more similar to those of juvenile females.

Though our observations followed expected social dynamics, assessments of social groups and corresponding association rates using GPS monitoring present a significant research challenge. Dyads with a combination of high VI (> 40%) and DI (> 0.15) can likely be considered members of the same social groups (Tosa et al. 2015). However, achieving a sufficient sample of deer representative of relevant sex and age classes requires an intensive and localized capture effort. Only 76 of 592 (12.8%) potential seasonal dyads in our study met these thresholds in our relatively large study area (Figure 1B), resulting in several demographic‐specific association rates that could not be assessed. Additionally, many deer monitored during our study died during winter, precluding our ability to compare winter contact rates with those observed during summer and rut. Ultimately, the inherently interactive effects of season and demographic class necessitate a large dataset from a localized area for adequate statistical assessment.

Most studies of CWD are limited to disease transmission implications rather than direct assessments due to challenges with pre‐mortem assessments of CWD infections (Bartz et al. 2024). As a result, we know of only two peer‐reviewed studies assessing movements of deer with known CWD infections (Skuldt et al. 2008; Edmunds et al. 2018). Deer infected with CWD were less active and had smaller home ranges than uninfected deer (Edmunds et al. 2018). Likelihoods of infected deer to explore, disperse, or migrate outside their home ranges, however, produced contradicting results. Deer from Wisconsin infected with CWD remained within their home ranges (Skuldt et al. 2008). White‐tailed deer (Edmunds et al. 2018) and mule deer ( *O. hemionus* ) (Barrile et al. 2024) from Wyoming infected with CWD migrated and dispersed in equal proportions to uninfected conspecifics. Deer with known CWD infections also interacted with scrapes similarly to healthy deer (Huang, Demarais, Strickland, et al. 2024). Our results indicated association rates among dyads inclusive and exclusive of CWD‐infected deer were similar. Barrile et al. (2024) indicated movements of CWD‐infected individuals were not altered until deer were within 6 months of death due to CWD. This may indicate that deer included in our analyses had not yet reached this clinical stage, at which point their association rates might change. As pre‐mortem assessments of CWD and other diseases improve, evaluations of contact rates among individuals with known infections are warranted.

## 4 Conclusions

Considering our initial questions, our review identified physical touch, social groups, spatial overlap, association rates, and social networks as principal themes underlying intraspecific contact among deer (Figure 2; Table 1). Visual observations were the primary field method used to assess touching and social group dynamics. These studies indicated deer–deer touching is infrequent (< 2 touches per hour), with high‐risk behaviors (e.g., grooming, tending) occurring primarily between adult females and their offspring during summer or males during rut. Comparatively, spatial overlap and association rates were primarily studied using VHF and GPS monitoring devices as applied in our case study. Spatial overlap correlates to direct and indirect association rates, and practical metrics for discerning group membership include high VI and DI values (VI > 40%; DI > 0.15). Estimates of association rates generally indicate high within‐group and low between‐group association rates.

Our case study addressed an important knowledge gap by examining season‐specific association rates across demographic classes (Table 3). A principal challenge when assessing disease transmission using GPS monitoring data is the inability to assess transmission probability during intraspecific contact (i.e., associations do not always equate to disease transmission). Acknowledging these limitations, intraspecific contact among deer was described similarly regardless of methodology and study intent. It is therefore likely that modern approaches using GPS monitoring provide accurate depictions of deer disease dynamics without the need for additional studies using visual observations, but concentrated monitoring efforts (i.e., large sample sizes in small areas) will provide greater insights than our case study. In support of analytical assessments of demographic‐specific contact rates, we provided a replicable analysis applied in our case study (Wehr and Walter 2025).

The prevailing consensus is that contact rates are much greater within social groups, with between‐group contact rates being generally low. However, adequate visual assessments required marking numerous deer to identify individuals (Nixon and Mankin 2007), and analyses of social group membership using modern movement ecology approaches remain difficult. Prior applications suggest that high spatial overlap may be adequate (Habib et al. 2011; Magle et al. 2013), while others contend that highly correlated movements should also be considered (Tosa et al. 2015, 2017). Our case study indicated a high degree of correlation between association rates, spatial overlap, and correlated movements; specifically, association rates among dyads with high spatial overlap were much higher than those with low spatial overlap. However, if only dyads with high spatial overlap and highly correlated movements are considered members of the same social group, intragroup transmission could be much higher than previously believed. This warrants further research, and social network analyses may be a useful solution to this problem. Existing social network analyses of deer were largely descriptive. Further, all studies identified by our review used static rather than dynamic social networks, which fail to reflect changes in association rates over time (Springer et al. 2017). Effective applications of social networks to deer included Koen et al. (2017)'s comparison of association rates in differing landscapes and identification of indirect transmission via male super‐spreaders during rut (Egan et al. 2023; Hearst et al. 2023). Combining social networks with contact rates in the context of seasonally dynamic deer social groups seems the logical next step to progress our understanding.

The underlying impetus of this work was to assess contact rates among demographic classes in support of disease modeling and deer management. Our literature review indicated contact rates follow well‐described patterns of deer behavior, suggesting managers may apply intuitive knowledge to understanding potential disease transmission dynamics. We did not, however, identify any comprehensive estimates of season‐specific, demographic‐specific contact rates that could be applied to modeling efforts in support of more advanced decision‐making approaches; our case study partially addressed this gap using association rates (Table 3). Our estimates should provide useful inputs for future disease transmission models, though future efforts could consider concentrating monitoring efforts to maximize observations of social associations (Davis et al. 2018). Prior research that could have incorporated our estimates includes meta‐population (e.g., Khadonova 2024) and agent‐based models (e.g., Belsare et al. 2020a) of CWD transmission. Such models can be used to inform specific management strategies (e.g., CWD monitoring strategies [Belsare et al. 2020b; Belsare and Stewart 2020] and influences of predators and other diseases on CWD dynamics [Khadonova 2024; Strasburg and Christensen 2024]), and our estimates are intended to support similar future endeavors.

### 4.1 Future Directions

Our review indicated several possible metrics for assessing association rates, and the *wildlifeDI* package can estimate most of these metrics (Long et al. 2014, 2022; Long 2024). We used the proportion of concurrent locations (*prox* in *wildlifeDI*) in our case study as the most intuitive value for association rates. However, the assessment of association rates using GPS monitoring data is an advancing field, and models offering potential improvements continue to be developed. Recently, the movement‐driven modeling of the spatial–temporal infection risk (MoveSTIR) framework was developed to make informed predictions of disease risk (Wilber et al. 2022). MoveSTIR considers direct–indirect associations on a continuum with disease risk encapsulated by the duration of time spent in spatially concurrent locations (Wilber et al. 2022). The corresponding metric calculable from MoveSTIR models is the transmission kernel, also known as the interaction kernel, which is a time‐weighted estimate of unidirectional transmission probability from one individual to another (Wilber et al. 2022). Yang et al. (2023) increased the replicability of MoveSTIR using continuous‐time movement models and applied it to mule deer and feral pigs ( *Sus scrofa* ). Herraiz et al. (2024) applied the transmission kernel and found generally greater within‐ than between‐species disease transmission kernels among ungulates in Spain.

As considered by MoveSTIR, indirect transmission is an important aspect of disease dynamics, but it remains understudied among deer. Our review identified assessments of potential indirect transmission at specific sites (i.e., scrapes [Hearst et al. 2021; Egan et al. 2023; Hearst et al. 2023] and agricultural feed [Wilber et al. 2019]) and via GPS monitoring (Schauber et al. 2007; Rustand 2010; Vargas Soto et al. 2025). However, the inferences drawn from these assessments were limited, warranting greater study of indirect associations from available GPS monitoring data. Recursion analysis may provide a useful avenue for future study. Recursive movements are those wherein an individual revisits the same location multiple times. Recursion analyses can identify sites with high potential for disease transmission (e.g., anthropogenic food sources [Mysterud et al. 2021]) or assess sites of known interest (e.g., bait sites [Roden‐Reynolds et al. 2022]), and the *recurse* R package facilitates such efforts (Bracis et al. 2018). Recursion analyses may also identify locations repeatedly used by multiple individuals allowing for identification of sites and, by extension, habitats where indirect transmission is most likely.

## Author Contributions

**Nathaniel H. Wehr:** data curation (lead), formal analysis (lead), investigation (equal), methodology (lead), visualization (lead), writing – original draft (lead), writing – review and editing (lead). **Kristin J. Bondo:** conceptualization (equal), investigation (equal), writing – review and editing (equal). **Christopher S. Rosenberry:** conceptualization (equal), funding acquisition (equal), investigation (lead), writing – review and editing (equal). **David Stainbrook:** investigation (equal), writing – review and editing (equal). **Bret D. Wallingford:** investigation (equal), writing – review and editing (equal). **W. David Walter:** conceptualization (equal), funding acquisition (equal), project administration (lead), supervision (lead), writing – review and editing (equal).

## Funding

This work was supported by the Pennsylvania Game Commission (Research Project Numbers 31 and 49) and U.S. Department of Agriculture Animal and Plant Health Inspection Service.

## Conflicts of Interest

The authors declare no conflicts of interest.

## Data Availability

The R code used to process the data was published by Wehr and Walter (2025). Data are permanently archived on Movebank.org and may be accessed via request to the Pennsylvania Game Commission. Data were made available to anonymous peer reviewers as an *rda* file during the peer review process.
